# Management of appendicitis during COVID-19 pandemic; short-term outcomes

**DOI:** 10.1177/0036933020956316

**Published:** 2020-09-02

**Authors:** Radhakrishnan Ganesh, James Lucocq, Neville Ogbonnia Ekpete, Noor Ul Ain, Su Kwan Lim, Al Alwash, Saira Bibi, Afshin Alijani

**Affiliations:** Specialty Registrar, Department of General Surgery, Ninewells Hospital, UK

**Keywords:** Appendicitis, COVID-19, antibiotic, CT scan

## Abstract

**Background and aim:**

COVID-19 pandemic has predisposed patients undergoing surgery to post-operative infection and resultant complications. Appendicitis is frequently managed by appendicectomy. After the onset of the pandemic, selected cases of appendicitis were managed with antibiotics which is a recognised treatment option. Our objective was to compare the management of appendicitis and post-operative outcomes between pre- and post-COVID-19.

**Methods:**

Ninety-six patients were identified from before the onset of the pandemic (November 2019) to after the onset of the pandemic (May 2020). Data were collected retrospectively from electronic records including demographics, investigations, treatment, duration of inpatient stay, complications, readmissions and compared between pre- and post-COVID-19 groups.

**Results:**

One hundred percent underwent surgical treatment before the onset of pandemic, compared with 56.3% from the onset of the pandemic. A greater percentage of patients were investigated with imaging post-COVID-19 (100% versus 60.9%; p < 0.00001). There was no significant difference in the outcomes between the two groups.

**Conclusion:**

CT/MRI scan was preferred to laparoscopy in diagnosing appendicitis and conservative management of uncomplicated appendicitis was common practice after the onset of pandemic. Health boards can adapt their management of surgical conditions during pandemics without adverse short-term consequences. Long term follow-up of this cohort will identify patients suitable for conservative management.

## Introduction

The Coronavirus (COVID-19) pandemic has resulted in the adoption of a change in the overall health care strategy that will reduce the spread of the virus. In the hospital setting several measures have been taken to avoid unnecessary procedures or environments where the risk of exposure is high.

Right iliac fossa pain with suspected appendicitis is a common cause for emergency general surgical admission.^1^ Appendicectomy is the preferred management for those patients with appendicitis, except in patients with significant co-morbidities or alternate patient preference.^2^ APPAC trial has shown that Computed tomography (CT)-proven uncomplicated acute appendicitis is not a surgical emergency and that antibiotic treatment is a safe first-line treatment for uncomplicated acute appendicitis.^3^ Conservative management with antibiotics for uncomplicated appendicitis has the risk of recurrent appendicitis, and ranges from 16% to 40% at one year after initial treatment.^3–5^

The ongoing COVID-19 pandemic spread to the United Kingdom in late January 2020. The first COVID-19 patient was diagnosed in our region in March 2020. Non-essential operations were cancelled at our institution from the 21st of March. Our approach to managing appendicitis was modified recognising the risks of COVID-19 infection, such as avoiding aerosol generating procedures - in line with international and national guidelines.^6,7^ Patients with uncomplicated appendicitis were managed with non-operative treatment if possible, after the onset of the COVID-19 pandemic.

Before the onset of the pandemic, patients with a clinical diagnosis of appendicitis went on to have diagnostic laparoscopy. Young female patients had pelvic ultrasound and a urine pregnancy test to rule out gynaecological causes for the symptoms, before subjecting to laparoscopy. For older patients, or in cases where the diagnosis was unclear, a CT scan of the abdomen and pelvis was conducted to confirm the diagnosis of appendicitis, and rule out other sinister causes with similar presentations common in elderly patients.^8^ CT scan is avoided in younger patients in view of the risk of radiation; younger aged patients also have greater years remaining in which a radiation-induced malignancy may develop.^9^

Clinical evaluation of patients has been modified since the onset of the COVID-19 pandemic. Adult patients admitted with a suspected diagnosis of appendicitis had a CT scan of the abdomen and pelvis irrespective of the age group, these patients also had a CT chest at the same time, to look for any signs of COVID-19 infection. The reason behind this approach is 1, to get a definite diagnosis, 2, to assess the severity of appendicitis^10^ and 3, to rule out an active COVID-19 infection of the lungs.^11^ Patients with normal CT scan findings were discharged early to reduce spread of the virus.

Patients with confirmed CT scan findings of appendicitis were assessed for the suitability of conservative management based on the likelihood of intra-abdominal contamination due to perforation of the appendix and abscess formation. Decisions regarding the management strategy were made by the admitting surgeon depending on the clinical condition of the patient, taking into account the radiological findings and blood investigations.

Patients who were treated with conservative management were discharged once symptoms had resolved, worsening advice was given on discharge, and follow-up arrangement was dependent on the decision of the admitting consultant. After appendicectomy, patients were not routinely followed-up.

In this paper, our objective was to evaluate the change in our practice of managing patients admitted with acute appendicitis, and to investigate short-term outcomes after the onset of pandemic. As a more conservative approach was more likely to be adopted during the pandemic, our aim was to look into the outcomes of those patients treated for appendicitis pre COVID-19 pandemic and during the pandemic in the short term.

## Method

Adult patients admitted to the acute surgical unit of our institution with a diagnosis of acute appendicitis between November 2019 and May 2020 were identified based on radiological diagnosis or operative findings. We collected data relating to demographics, co-morbidities, inflammatory markers and radiological findings for these patients. We recorded the management strategy for each patient and followed each patient up for thirty-days looking for post-operative complications, readmissions, further imaging and intervention. Data was collected retrospectively using electronic records (Clinical Portal, Integrated Clinical Environment and Picture Archiving and Communication System).

At our institution, surgical practice changed from the 21st of March, marking the beginning of the post-COVID-19 group in this study. Short term outcomes were compared between those managed by surgical treatment in the pre-COVID-19 period versus those managed with selective conservative and surgical treatment during the pandemic period. Diagnostic methods (laparoscopy versus imaging) and management strategy (operative or non-operative) were recorded during the two time periods. Duration of stay, use of antibiotics, complications and the rate of readmission were compared between pre- and post-COVID-19 groups. Comparisons were made using Fisher’s exact test, Chi-Squared, and Mann-Whitney U tests using the STATA IC 16.1 statistical package.

## Results

A total of 96 adult patients (median age, 36; range, 14–80; M:F, 1.0:1.09) were identified from November 2019 to May 2020 (Table 1). Patients had either laparoscopy or imaging (CT scan & Magnetic resonance imaging (MRI) confirming the diagnosis of appendicitis. Eleven patients had co-morbidities, including diabetes mellitus, ischemic heart disease, hypertension, hypothyroidism, epilepsy, COPD and asthma. All patients were fit for surgical intervention based on the assessment of the surgical consultant in charge of the patient.

**Table 1.** Demographic data.

	Pre-COVID-19 (n = 64)	Post-COVID-19 (n = 32)	p-value
Median age, years (range)	37 (14–80)	37 (15–74)	0.45
Male:Female ratio	1.5:1.0	0.6:1	0.04
Patients with comorbidities, %	14.1	12.5	0.83
Median hospital stay, days (range)	2 (1–34)	3 (1–8)	0.09
Median admission WBC, ×10^9^/L, (range)	12.9 (4.3–24.4)	13.2 (6.2–18.9)	0.95
Median admission CRP, mg/L, (range)	82 (4–408)	69 (4–339)	0.95
Median duration of antibiotics, days (range)	7 (1–21)	7 (1–33)	0.12

CT scan was performed in 67.7% (65/96) and one patient had MRI (1.0%). Twelve (12.5%) patients had ultrasound, four of which also has a CT scan.

Eighty-two patients (85.4%) had appendicectomy and the remaining 14.6% (14/96) patients were managed conservatively with antibiotics. Seven patients (7.3%) underwent an open appendicectomy, five of where were converted from a laparoscopic approach.

### Pre-COVID-19 versus post-COVID-19

Sixty four (66.7%; median age, 37; range, 14–80; M:F, 1.5:1.0) were identified with a diagnosis of appendicitis from November 2019 until the change in management strategy at the onset of pandemic (21st March). Thirty-two patients (33.3%; median age, 36; range, 15–74; M:F, 1.0:1.7) were identified from the onset of the pandemic time until 15th May, 2020.

Pre-COVID-19, 60.9% of patients (39/64) had imaging prior to appendicectomy. One hundred percent (64/64) of patients had an appendicectomy for acute appendicitis before the change in our practice (Table 2). One patient had the procedure abandoned and later had an interval appendicectomy. Five patients had an open appendicectomy, three of which were converted from a laparoscopic approach.

**Table 2.** Pre and post-COVID-19 management of appendicitis and outcome.

	Pre-COVID-19 (n = 64)	Post-COVID-19 (n = 32)	p-value	Post-COVID-19 Odds Ratio (95% CI)
Investigation with CT/MRI, %	60.9 (39/64)	100 (32/32)	<0.00001	13.2000 (1.6619 to 104.8416)
Investigation with CT/MRI, % (patients <40 years of age)	30.6 (11/36)	100 (19/19)	<0.00001	43.3333 (5.1926 to 361.6265)
Surgery, % (number of patients)	100 (64/64)	56.3 (18/32)	<0.00001	0.0195 (0.0024 to 0.1572)
Surgery, % (number of patients) (patients <40 years of age)	100 (36/36)	52.6 (10/19)	<0.00001	0.0297 (0.0034 to 0.2586)
Antibiotics, % (number of patients)	70.3 (45/64)	100 (32/32)	0.0112	14.3478 (1.8331 to 112.2997)
Readmissions over 30-day follow-up % (number of patients)	12.5 (8/64)	9.3 (3/32)	0.67	0.7241 (0.1785 to 2.9384)
Readmission imaging, % (number of patients)	4.7 (3/64)	6.2 (2/32)	0.93	1.3556 (0.2149 to 8.5510)

In calculations of the Odds Ratio, in cases where there was no integer value (where cases are 0), all values in that calculation have been increased by 1 to allow statistical analysis.

Post-COVID-19, 100% (32/32) of patients had imaging, 31 with CT scan and one with MRI. None of the patients had positive findings on CT chest done during the post-COVID-19 period. Eighteen out of thirty-two patients (56.3%) of patients had an appendicectomy. Among them two patients had laparoscopy converted to open procedure. One patient was initially managed with laparoscopic washout and drain placement. This patient went on to have laparoscopic appendicectomy for perforated appendix during the same admission. The remaining 43.7% (14/32) patients were treated with antibiotics.

A greater percentage of patients were investigated with imaging post-COVID-19 versus pre-COVID-19 (100% versus 60.9%; p < 0.001) The percentage of patients treated surgically reduced from the onset of COVID-19 (p < 0.00001). In the group of patients <40 years of age, 30.6% of patients had imaging pre-COVID-19 versus 100% of patients post-COVID-19 (p < 0.0001) and 100% of patients underwent surgical intervention versus 52.6%. There was no statistical difference in the rate of readmissions between groups.

In the pre-COVID-19 period, post-operative ileus, collection requiring radiologically placed drain, and laparoscopic washout and adhesiolysis were causes of complicated admission and delayed discharge. There were no complications among patients managed conservatively or by operative treatment during the post-COVID-19 period.

#### Surgery versus conservative management

Eighty two patients were treated by surgical treatment (Median age, 37; range, 14-80; M;F, 1.34:1.0) starting from November 2019. Fourteen patients were treated conservatively with antibiotics (Median age, 24; range, 15-74; M;F, 1.0:2.5), all of which were in the post-COVID-19 period. Diagnosis of all those treated conservatively was made from imaging (CT scan & MRI). Among those treated conservatively, there were more female patients. No complications were recorded in those patients treated with non-operative management. One patient who was managed with antibiotics was readmitted with abdominal pain and was managed conservatively after CT scan did not show any complications.

## Discussion

A conservative approach was adopted in managing an acute surgical condition recognising the risk of increased post-operative morbidity and mortality in COVID-19 infected patients.^6^ All necessary precautions were taken during this period to avoid exposure to the virus. Steps were also taken to avoid patients coming to the hospital. Those attending hospital for various reasons were discharged early after managing them appropriately.

After the onset of COVID-19 pandemic, a conservative approach was adopted at our institution when possible in treating acute appendicitis, in line with the national guidelines.^7^ Clinical assessment including blood tests and radiological investigations with CT scan were used to diagnose and manage appendicitis. All patients were commenced on antibiotic treatment once a diagnosis was made. Decision regarding operative or conservative management was made by admitting surgeon based on signs of peritonism, sepsis and persistent symptoms.

Appendicitis is a clinical diagnosis and imaging is helpful in older patients to rule out sinister causes of symptoms.^8^ Before the onset of COVID-19 pandemic, our practice was to use CT selectively in diagnosing appendicitis and ruling out sinister causes with similar presentation in older patients. After the age of forty years the incidence of diverticulosis and malignancy increases which can present acutely with right iliac fossa pain.^12^ Laparoscopy was preferred in younger patients with suspected clinical diagnosis of appendicitis. CT scan is routinely used in some institution for all adult patients to confirm a clinical diagnosis of appendicitis. CT scan has high accuracy in diagnosing appendicitis and is used to assess the severity of appendicitis.^13,14^ Early appendicitis without intra-peritoneal contamination may be suitable for non-operative treatment with antibiotics.

CT scan is avoided in younger patients in view of the risk of radiation,^15^ however, laparoscopy is an invasive procedure and carries the risk of iatrogenic injury and anaesthetic complications.^16^ CT scan will also reduce the incidence of negative laparoscopy. In the present study, all patients under the age of 40 years underwent CT scan to diagnose appendicitis after the onset of COVID-19 pandemic. The risk of radiation exposure may not be significant considering that they do not require repeated scanning. MRI scan can be utilised in younger (less than 20 years) and pregnant patients to avoid radiation exposure in these patients.^2^ The role and risk of CT/MRI scan in diagnosing appendicitis should be discussed while consenting the patients for diagnostic laparoscopy.

The reported sensitivity for ultrasound varies in the literature (71–94%) and is lower than CT scan.^2^ Ultrasound is most often the first line imaging modality for suspected appendicitis. Sensitivity of ultrasound is operator dependent. Swollen appendix was noted in seven of the twelve patients examined. Ultrasound was also used to rule out other causes of symptoms mostly in female patients.

Adoption of a non-operative strategy and selective management with surgical treatment during the pandemic period may throw light on suitability of antibiotic treatment in patients with early appendicitis. Retrospective analysis of the outcome of conservative management at a later date will help in the grading of CT scan findings in selecting patients for non-operative treatment. This grading can be validated with a future prospective study.

This is not a randomised control study to assess the superiority of one modality of treatment over other; however, follow up of the patients with uncomplicated appendicitis treated with antibiotics will give an opportunity to understand the natural course of this disease. The role of antibiotics in halting the progress of this disease to a complicated appendicitis and recurrence may be studied by close follow-up of these patients. Identifying the risk factors for recurrence will help us in selecting patients who can be treated safely with antibiotics. Use of antibiotics has its own risk including clostridium difficile infection and emergence of resistant organisms.^17,18^

Patients were followed-up for a minimum period of 30 days. Long-term follow-up is required to evaluate long-term outcomes and will inform us the long-term effect of pandemics on surgical practice. Studies have shown that among patients treated with antibiotics, 17–64% required operation within 30 days of presentation.^3,4,19,20^ In our cohort, 0% of patients required operation over the 30 days follow-up.

At our institution, patients who were managed conservatively had uncomplicated appendicitis with little evidence of intra-peritoneal contamination. Long-term outcomes (e.g. 1 year) will specifically demonstrate whether or not conservative management is a safe treatment option for uncomplicated appendicitis. The same patient group will be evaluated in one year to investigate for complications, further readmission and intervention.

## Conclusion

From the onset of the COVID-19 pandemic, patients with acute appendicitis were managed safely with good outcomes. Acute uncomplicated appendicitis was frequently managed non-operatively with antibiotic treatment. Patients with significant co-morbidities, those with local or general contamination, and those who failed medical management went on to have appendicectomy. Health boards can successfully adapt their management of surgical conditions, such as appendicitis, during pandemics without negative short-term outcomes. Long term follow-up of these patients will be useful in deciding the role of non-operative management in treating uncomplicated appendicitis.
